# Prenatal and postnatal influences on behavioral development in a mouse model of preconceptional stress

**DOI:** 10.1016/j.ynstr.2024.100614

**Published:** 2024-02-03

**Authors:** Joseph Scarborough, Monica Iachizzi, Sina M. Schalbetter, Flavia S. Müller, Ulrike Weber-Stadlbauer, Juliet Richetto

**Affiliations:** a Institute of Pharmacology and Toxicology, University of Zurich-Vetsuisse, Zurich, Switzerland; b Neuroscience Center Zurich, University of Zurich and ETH Zurich, Zurich, Switzerland

**Keywords:** Preconception stress, Pregnancy, Cross-fostering, Behavior

## Abstract

Depression during pregnancy is detrimental for the wellbeing of the expectant mother and can exert long-term consequences on the offspring's development and mental health. In this context, both the gestational environment and the postpartum milieu may be negatively affected by the depressive pathology. It is, however, challenging to assess whether the contributions of prenatal and postnatal depression exposure are distinct, interactive, or cumulative, as it is unclear whether antenatal effects are due to direct effects on fetal development or because antenatal symptoms continue postnatally. Preclinical models have sought to answer this question by implementing stressors that induce a depressive-like state in the dams during pregnancy and studying the effects on the offspring. The aim of our present study was to disentangle the contribution of direct stress *in utero* from possible changes in maternal behavior in a novel model of preconceptional stress based on social isolation rearing (SIR). Using a cross-fostering paradigm in this model, we show that while SIR leads to subtle changes in maternal behavior, the behavioral changes observed in the offspring are driven by a complex interaction between sex, and prenatal and postnatal maternal factors. Indeed, male offspring are more sensitive to the prenatal environment, as demonstrated by behavioral and transcriptional changes driven by their birth mother, while females are likely affected by more complex interactions between the pre and the postpartum milieu, as suggested by the important impact of their surrogate foster mother. Taken together, our findings suggest that male and female offspring have different time-windows and behavioral domains of susceptibility to maternal preconceptional stress, and thus underscore the importance of including both sexes when investigating the mechanisms that mediate the negative consequences of exposure to such stressor.

## 1 Introduction

Pregnancy is a major event in a woman's life that encompasses many psychological, biological and life-style changes. While being without compilations for some expecting mothers, 10–20% of pregnant women meet the criteria for depression (Fisher et al., 2012; Howard et al., 2014; Melville et al., 2010), a number that increases to 30% when relying on standardized self-report data (Melville et al., 2010). Importantly, perinatal mental disorders such as maternal depression contribute substantially to societal and health burdens, as they not only affect the mother, but also can induce negative effects on the offspring's mental health (Pawlby et al., 2009). Indeed, if left untreated, maternal depression and related mood disorders can have a variety of detrimental consequences for the newborn (Ashman et al., 2002; Davalos et al., 2012; Glover, 2014; Hammen and Brennan, 2003), including increased infant morbidity and neurodevelopmental abnormalities (Davalos et al., 2012; Gentile, 2017; Nulman et al., 2012; Rogers et al., 2020), as well as increased suicidal risk in the mother (Admon et al., 2021).

In the context of maternal perinatal depression, both the gestational environment and the postpartum milieu may be negatively affected by the depressive pathology. It is, however, difficult to separate the relative contribution of prenatal versus postnatal maternal factors in terms of their causal impact on the offspring. Specifically, it is challenging to assess whether the contributions of prenatal and postnatal depression exposure are distinct, interactive, or cumulative, as it is unclear whether antenatal effects are due to direct effects on fetal development or because antenatal symptoms continue postnatally. Maternal depression may, indeed, affect fetal neurodevelopment before birth via altering the intrauterine environment, and it may also lead to poor maternal–infant attachment after birth, thus influencing the infant's development postnatally. Besides others, depression may lead to alterations of the maternal immune system and HPA axis (Osborne et al., 2018), alterations in placental microenvironment, and indirect effects mediated by maternal risk-taking behaviors such as substance abuse, inadequate nutrition and reduced compliance with prenatal care (Newman et al., 2016; Sawyer et al., 2019; Biaggi et al., 2016). In the postpartum period, a mother's attachment to her baby can be negatively influenced by depressive symptoms, resulting in difficulty bonding and poor mother-infant relationship (Feldman et al., 2009; Stein et al., 2014). In turn, the infant's early attachment relationship with its mother has a strong impact on its emotional regulation throughout development into adulthood, with poor attachment predisposing to behavioral difficulties later in life (Stein et al., 2014). Lastly, the picture is complicated by the fact that prenatal depression is one of the leading risk factors for post-partum depression (Leigh and Milgrom, 2008; Norhayati et al., 2015), and one is often thus followed by the other.

Against this background, preclinical models have attempted to disentangle the contribution of direct stress *in utero* from possible changes in maternal behavior resulting from the depressive-like state present in the dams. While such models cannot, of course, fully recapitulate human maternal depression, they rely on the application of various different stressors during pregnancy to elicit a behavioral phenotype in the dams which is relevant to the human disease. For example, cross-fostering studies have been mainly conducted in the prenatal stress model, which is often used as a proxy to model certain aspects of prenatal maternal depression. In such studies, prenatally stressed offspring are separated from their mother and reared by a non-stressed foster mother, and vice-versa. The outcomes of such studies are mixed, with reports suggesting a greater impact of alterations in postnatal maternal care on adult offspring behavior (Barros et al., 2006; Golub et al., 2016; Hauser et al., 2009; Schmidt et al., 2018; Feng et al., 2021), while others imply a more profound effect of prenatal stress on the offspring's development (Nyirenda et al., 2001; Yang et al., 2006; Jiao et al., 2013). Such discrepancies are likely due to differences in timing and specifics of the different stress paradigms, rendering comparisons across studies challenging. Moreover, prenatal stress models, *per se*, present some caveats when modelling the clinical scenario, in that they do not recapitulate one of the main eliciting factors of human depression, namely social stress.

The aim of the present study was to expand previous findings to encompass a novel and clinically-relevant model of preconceptional stress that is elicited by social deprivation. Specifically, the model is based on the social isolation rearing (SIR) paradigm applied to nulliparous female mice prior to mating in order to induce a depression-like state that is initiated before conception and continues throughout gestation (Scarborough et al., 2021). Thus, a key strength of this model is its etiologically-relevant basis and ontopathogenic validity, as depression in humans is believed to be elicited by social stress rather than physical stress (Bekhet et al., 2008; Blazer and Hybels, 2005; Heinrich and Gullone, 2006). We have previously shown that the SIR paradigm is effective in eliciting alterations in affective, social and anxious behaviors, together with physiological changes such as weight gain and altered basal corticosterone levels, all of which are relevant to the human depression (Scarborough et al., 2021).

As the depressive-like state is not limited to a specific time window during pregnancy, this model attempts to model the most clinically challenging cases in which depression is present before and/or throughout the whole of pregnancy, as opposed to post-partum or during specific stages of pregnancy only (Pawlby et al., 2009; Plant et al., 2016). In addition, we identified a variety of behavioral and transcriptomic abnormalities in male and female offspring born to socially isolated mothers, which could partly be prevented by maternal fluoxetine (FLX) treatment (Scarborough et al., 2021).

Our present study builds on these previous findings and investigates whether the alterations observed in SIR offspring are the result of direct *in utero* alterations or if they are driven by abnormalities in maternal behavior resulting from the depressive-like state present in the dams. Interestingly, we uncover subtle changes in the maternal behavior of SIR dams, who spend less time on the nest, and licking and grooming their pups, during the resting phase. Despite such alterations in the postnatal environment, we observe marked sex-dependent differences when analysing offspring behavior and transcriptional signatures in the prefrontal cortex (PFC) and amygdala (AMY). Indeed, male offspring are more sensitive to the prenatal environment, as demonstrated by behavioral and transcriptional changes driven by their birth mother, while females are likely affected by more complex interactions between the pre and the postpartum milieu, as suggested by the important additional impact of their surrogate foster mother.

## 2 Methods

### 2.1 Animals

C57BL6/N mice (Charles River Laboratories, Sulzfeld, Germany) were used throughout the study and maintained in an animal vivarium. All procedures described in the present study had been previously approved by the Cantonal Veterinarian's Office of Zurich, and all efforts were made to minimize the number of animals used and their suffering. C57BL6/N mice were housed in individually ventilated cages (IVCs) in a specific-pathogen-free (SPF) holding room, which was temperature- and humidity-controlled (21 ± 3 °C, 50 ± 10%), and kept under a reversed light–dark cycle (lights off: 07:00 a.m.–07.00 p.m.). All animals had ad libitum access to the same food (Kliba 3436, Kaiseraugst, Switzerland) and water throughout the entire study. Animals were handled and monitored by the experimenters three times per week, prior to and throughout testing, to ensure the animals were habituated to handling.

### 2.2 Social isolation paradigm

Female mice at postnatal day (PND) 21 were either housed individually (SIR) (N = 20) or group housed (GRP) (N = 16) for 10 weeks until they were subjected to selected behavioural testing (further details below), followed by a timed mating procedure. A detailed summary of the allocation of animals, litters and offspring to the various experimental manipulations is provided in Supplementary Table S1.

### 2.3 Timed mating procedure

Timed-pregnant animals were generated via on-site breeding, which began after the 10 weeks behavioural testing. To this end, 20 SIR and 16 GRP female animals deriving from the above-mentioned groups were subjected to a timed-mating procedure as previously described (Meyer et al., 2005; Mueller et al., 2018). Males included in the mating procedure were previously left undisturbed in group housing. Successful mating was verified by the presence of a vaginal plug, upon which dams that had previously been socially isolated were housed individually throughout gestation, while dams that had previously been group housed were housed in groups of two up until 2 days prior to delivery of the pups, in order to keep each litter as a single statistical unit.

### 2.4 Cross-fostering and weaning of the offspring

Pregnant females were closely monitored on the days immediately prior to their predicted birth windows to ensure the correct birthdates of the litters. Cross-fostering was performed as described previously (Schwendener et al., 2009). Briefly, on PND1, offspring born to SIR and GRP animals were cross-fostered to surrogate rearing mothers. In detail, part of a given litter was allocated to a SIR surrogate rearing mother and part to a GRP rearing mother. Each surrogate mother thus simultaneously fostered pups originating from both prenatal housing conditions, but not its own offspring. SIR and GRP offspring were marked on the left and right hind paw, respectively. A total of 22 litters (11 SIR and and 11 GRP) were cross-fostered to 22 rearing mothers (11 SIR and and 11 GRP).

The cross-fostering resulted in four experimental treatment groups (see Supplementary Fig. S1): (1) offspring birthed by a GRP housed mother and raised by a GRP housed mother (GRP-pre/GRP-post), (2) offspring birthed by a GRP housed mother and raised by a SIR housed mother (GRP-pre/SIR-post), (3) offspring birthed by a SIR housed mother and raised by a GRP housed mother (SIR-pre/GRP-post), and (4) offspring birthed by a SIR housed mother and raised by a SIR housed mother (SIR-pre/SIR-post). Offspring were weaned on postnatal day (PND) 21. Littermates of the same sex were caged separately and maintained in groups of 3–5 animals per cage. They were kept in the same vivarium and caging systems (IVCs) and were maintained under a reversed light–dark cycle and ad libitum food access as described above.

### 2.5 Assessment of maternal behavior

Starting on postnatal day (PND) 1 and running to PND 7, maternal behaviour was assessed visually from the outside of the cage 6 times daily. We focused on this period because maternal observations over the first week postpartum are expected to be most critical for the reliable assessment of possible differences in maternal behavior (Schwendener et al., 2009). Monitoring occurred every 4 h, and the mother's behavior was scored for 60 s at 5 timepoints within the 30 min observation period. Maternal behavior was subdivided into the following categories, (see further details in the supplementary material) adapted from (Macri et al., 2004) and (Ruedi-Bettschen et al., 2004): Licking/grooming; Self-licking/-grooming; Eating/drinking; Nursing; Nest-building; Off nest. Maternal behavior was analysed separately in the dark/active phase and in the light/resting phase.

### 2.6 Female and offspring behavioral testing

Females, after 10 weeks of SIR, and offspring, from PND 70, were subjected to selected behavioral testing. Specifically, the females were testes in the light dark box, open field, and social interaction tests after 10 weeks of SIR and before mating. Their offspring were left undisturbed until PND 70, after which both female and male animals were subjected to an elevated plus maze test, a social interaction test, a Y-maze spatial recognition test and a temporal order memory test. The same testing order was always applied to each animal, with 3–4 testing-free resting days being imposed between each test. Behavioral testing commenced roughly 2 h after onset of the dark phase (9AM) and continued throughout the dark phase up until 2 h before the onset of the light phase (5PM). Detailed description of the behavioral testing procedures is provided in the Supplementary Material.

### 2.7 RNA extraction and quantitative real-time PCR analyses

The adult offspring were killed by decapitation under Isoflurane anaesthesia 48–72 h after completion of behavioral testing. The brains were rapidly extracted from the skull (within <20 s) and placed on an ice-chilled plate. Coronal sections were prepared using razorblade cuts along the following coordinates with respect to bregma: anterior–posterior +2.0 to +1.0, +1.0 to 0.0, 0.0 to −1.0, −1.0 to −2.0, and −2.0 to −3.0 mm. Discrete brain regions were then collected using a micropunch needle (1 mm in diameter) generating micropunches of distinct brain areas as described previously (Weber-Stadlbauer et al., 2017). Specifically, the micro-punching of the AMY encompassed the collection of the central and basolateral nuclei with reference to bregma (−1.2 to −2.2 mm), while the PFC specimens included the anterior cingulate, and the prelimbic and dorsal parts of the infralimbic subregions (bregma: +2.0 to +1.0 mm). The rational for including the PFC and AMY is twofold: on one hand, it is based on their critical involvement in regulating behavioral functions investigated in this study, such as emotional processing, anxiety and cognition (Dolan, 2002; Phelps and LeDoux, 2005). On the other hand, we wanted to build on and expand previous transcriptional findings that we had obtained in SIR offspring (Scarborough et al., 2021).

mRNA was extracted with the SPLIT kit (Lexogen, Germany) according to manufacturer's recommendations and qPCR was performed as described in the supplementary information.

Custom-designed probe and primer sequences, or product codes, used for the various genes of interest and reference gene (36B4) are summarized in Supplementary Table S2 and were purchased from Eurofins Genomics GmbH (Germany) and from Thermo Fisher Scientific (Germany). The target genes were selected based on transcriptomic changes already identified in the SIR model (Scarborough et al., 2021), to assess whether such SIR-induced transcriptomic changes were due to prenatal or postnatal factors.

### 2.8 Statistical analyses

All data met the assumptions of normal distribution and equality of variance; and all data were analysed using parametric analysis of variance (ANOVA) or Student's T-Test. Whenever appropriate, ANOVAs were followed by Tukey's post-hoc test to control for multiple comparisons. All statistical analyses were performed using SPSS Statistics (version 22.0, IBM, Armonk, NY, USA) and Prism (version 7.0; GraphPad Software, La Jolla, CA, USA). Statistical significance was set at *p* < 0.05.

### 2.9 Blinding and randomization

Throughout the behavioral testing, maternal behavior scoring, and molecular investigations, the experimenters were blind to the experimental groups of the animals. The testing order of the mice was randomized prior to testing, and females were allocated to either GRP or SIR housing with balanced randomization (to reach the desired number of animals per group).

## 3 Results

### 3.1 Effects of SIR during preconception on dam behavior and postpartum maternal behavior

**Fig. 1:**
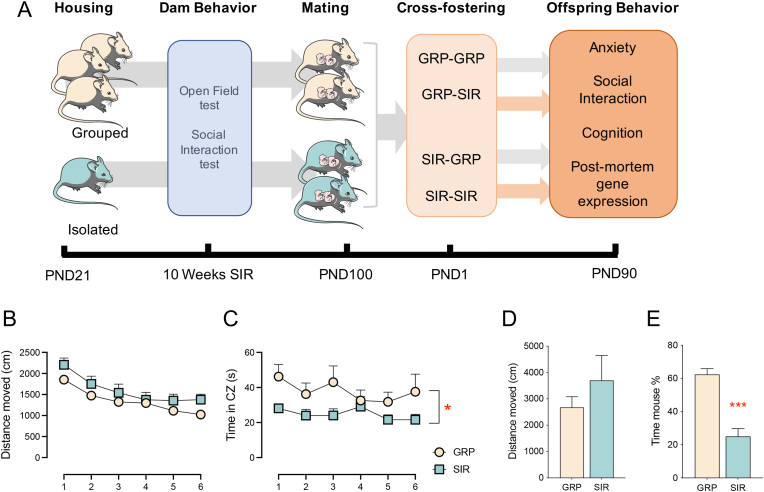
**Experimental paradigm and behavioral readouts in SIR and GRP-housed female mice**. Physiological and behavioral effects of SIR in female mice prior to conception. (A) Graphical representation of the experimental paradigm. C57BL6/N female mice were housed in social isolation or in groups from PND21 onwards. After 10 weeks of social isolation or group housing, animals were subjected to behavioral testing in the open field test and social interaction test. The animals were then exposed to a timed mating procedure and to a cross-fostering paradigm at birth. Offspring were then left undisturbed, weaned into same-sex grouped cages and subjected to behavioral testing in the elevated plus-maze test, the social interaction test, the Y-maze test of spatial recognition memory, and the temporal order test. (B) The line plot depicts the total distance moved in the open field test at the 10 weeks testing timepoint. (C) The line plot depicts the time spent in the center zone in the open field test at the 10 weeks testing timepoint (SIR vs GRP *p < 0.05). (D) The bar plot depicts the total distance moved during the social interaction test at the 10 weeks testing timepoint. (E) The bar plot depicts the % exploration time of an unfamiliar mouse compared to a dummy object during the social interaction test at the 10 weeks testing timepoint (SIR vs GRP ***p < 0.0001). N = 20 SIR and 16 GRP animals per group. All values are means ± S.E.M.

First, we confirmed the depressive-like phenotype induced by SIR in female mice prior to conception. Hereby, we focused on two main behavioral alterations induced by this housing regimen, namely anxiety and reduced social interaction (Scarborough et al., 2021). In line with our previous results (Scarborough et al., 2021), SIR females spent significantly less time interacting with a live mouse compared to a dummy in the social interaction test (t_(34)_ = 6.089, *p* < 0.0001, Fig. 1E), which was not confounded by differences in locomotor activity (Fig. 1D). Moreover, SIR mice also displayed increased anxiety-like behavior in the open-field test, as evident by the reduced time spent in the center zone compared to GRP mice (F_(34)_ = 5.233, *p* = 0.0285, Fig. 1C). There were no overall differences in locomotor activity in the open field test (Fig. 1B). In addition, SIR females also gained more weight over time compared to GRP animals (Supplementary Fig. S2), as shown previously (Scarborough et al., 2021).

**Fig. 2:**
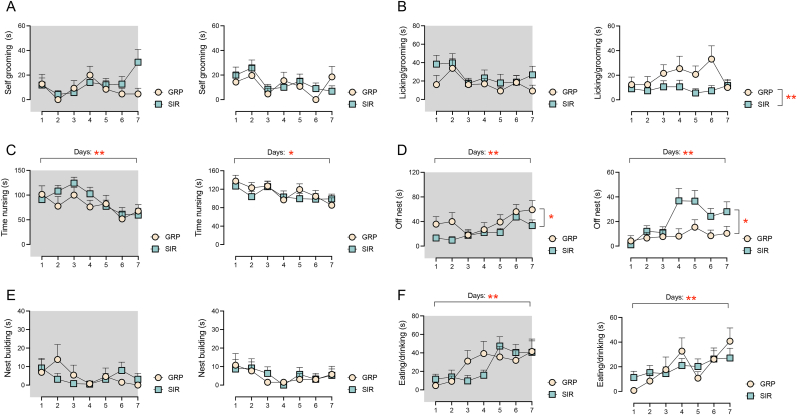
**Effects of SIR on postpartum maternal behavior in the resting and active phases.** Maternal behavior was assessed from postnatal days 1–7. The line plots depict (A) Self-grooming, (B) Licking/grooming, (C) tTime nursing, (D) Off nest, (E) Nest building, (F) Eating/drinking. The assessment of maternal behavior was performed during the dark/active phase (in gray) and during the light/resting phase (in white). N = 19 SIR and 13 GRP animals per group. All values are mean ± S.E.M. *p < 0.05, **p < 0.01.

We next assessed whether the depressive-like state induced by SIR impacted maternal behavior postpartum. The absolute times dedicated to each individual maternal behavior were analysed using 2 × 7 (prenatal treatment x days) RM ANOVAs, and each analysis was performed separately for the active (dark) phase and the resting (light) phase. Prenatal SIR led to a significant decrease in time spent licking and grooming the pups during the resting phase, as supported by the significant main effect of SIR (F_(1,30)_ = 8.8, p < 0.01; Fig. 2B). Concomitantly, prenatal SIR led to a significant overall increase, and decrease, of time spent off nest during the resting and active phases, respectively (main effect of housing; active phase: F_(1,30)_ = 6.6, p < 0.05; resting phase: F_(1,30)_ = 5.8, p < 0.05; Fig. 2D). For both experimental groups, contact with the pups was highest in the initial days postpartum, and gradually reduced afterwards, leading to a main effect of days on time off nest in both the active and resting phases (active phase: F_(6,150)_ = 3.7, p < 0.01; resting phase: F_(6,150)_ = 4.8, p < 0.01; Fig. 2D). Maternal SIR did not exert any significant influence on eating/drinking, self-grooming, nursing and nest building (Fig. 2A–C, E, F). In general, pup nursing was highest in the initial days postpartum and subsided afterwards, whilst eating/drinking reached maximal levels between PND 5 and PND 7 (Fig. 2C–F), in both the active and resting phases. This pattern of results led to significant main effects of days in the ANOVAs of these measures (nursing active: F_(6,150)_ = 4.1, p < 0.01; nursing resting: F_(6,150)_ = 3.2, p < 0.05; eating/drinking active: F_(6,150)_ = 4.6, p < 0.01; eating/drinking resting: F_(6,150)_ = 3.8, p < 0.01). Of note, SIR did not affect litter sizes, which were comparable across both groups (Supplementary Fig. S3).

### 3.2 Prenatal and postnatal effects of maternal SIR on offspring behavior

**Fig. 3:**
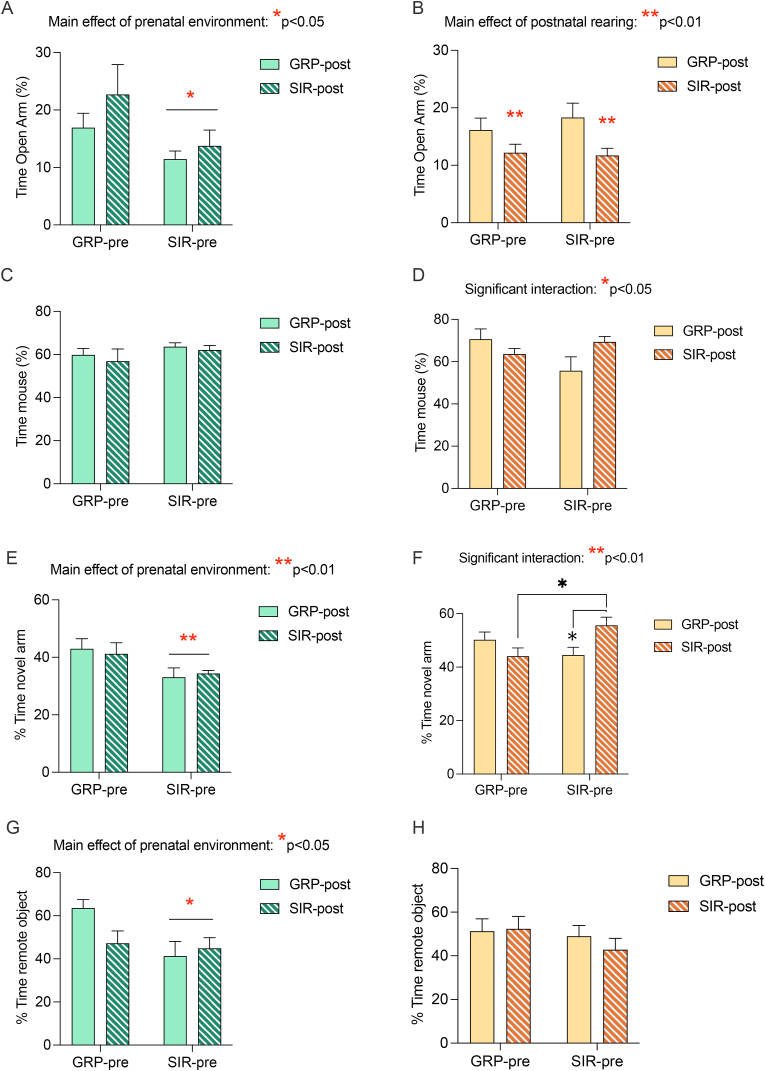
**Effects of pre- and postnatal maternal factors on behavior and cognition in the offspring**. From PND 70 onwards, adult offspring were subjected to behavioral and cognitive testing in order to explore whether prenatal or postnatal maternal factors had greater impact on the offspring's behavior. (A, B) the bar plots depict the % time (as a function of total exploration time) spent in the open arms of the elevated plus maze in male (green hues) and female (yellow hues) offspring, respectively; (C, D) the bar plots depict the % exploration time (as a function of total exploration time) of an unfamiliar mouse compared to a dummy object in the social interaction test in male and female offspring, respectively; (E,F) the bar plots depict the % exploration time (as a function of total exploration time) of the novel arm in the Y-maze test of spatial recognition memory in male and female (Post-hoc comparisons: GRP-pre/SIR-post vs SIR-pre/SIR-post *p < 0.05, SIR-pre/GRP-post *vs* SIR-pre/SIR-post *p < 0.05) offspring, respectively; (G,H) the bar plots depict the relative time spent exploring the remote object (as a function of time spent exploring both remote and recent objects) in the temporal order test in male and female animals. N = 9–12 male animals per group, N = 11–14 female animals per group. Significant main effects and interactions uncovered by the 2-way ANOVA are depicted with red asterisks, while significant post-hoc comparisons are depicted with black asterisks. Prenatal conditions are grouped on the x-axis (GRP-pre: offspring born to a GRP dam; SIR-pre: offspring born to a SIR dam), while the colour of the bars indicate the postnatal rearing conditions (GRP-post: offspring raised by a GRP dam; SIR-post: offspring raised by a SIR dam). All values are mean ± S.E.M.

We went on to perform behavioral characterizations in the adult cross-fostered offspring to disentangle direct effects of SIR *in utero* from possible changes in the dam's maternal behavior resulting from SIR. As depicted in Fig. 3, we replicated our previous findings of increased anxiety-like behavior and cognitive impairments in SIR offspring. Interestingly, however, we observed striking sex-dependent effects when considering the relative prenatal and postnatal maternal contributions to these alterations.

In male offspring, the behavioral outcome was largely driven by the prenatal environment, with seemingly no effect of the immediate postnatal environment. Specifically, in the EPM, male offspring of SIR females spent significantly less time in the open arm compared to offspring of GRP mothers, regardless of whether they were raised by a SIR or GRP foster mother (main effect of prenatal environment: F_(1, 39)_ = 5.5, *p* = 0.02; Fig. 3A). A similar pattern was observed in both the Y-maze test of spatial recognition memory and in the temporal order memory test. Indeed, male SIR offspring displayed a marked reduction in the relative time spent in the novel arm, and spent significantly less time interacting with the remote object, respectively, when compared to male offspring of GRP animals. Once again, these effects were completely irrespective of the foster mother, as demonstrated by the main effects of prenatal environment in the Y-maze test (F_(1, 39)_ = 7.7, *p* = 0.008 Fig. 3E) and TOMT (F_(1, 39)_ = 5.0, *p* = 0.03; Fig. 3G). No effects were observed in social interaction, in line with previous findings (Scarborough et al., 2021).

On the other hand, the interplay between prenatal and postnatal factors in mediating female offspring behavior appeared more complex. In the EPM, female behavior was mainly driven by postnatal factors, with female offspring fostered to a SIR mother, regardless of the birth mother, spending significantly less time in the open arm compared to offspring fostered to a GRP mother (main effect of postnatal rearing: F_(1, 47)_ = 7.7, *p* = 0.007, Fig. 3B). When considering cognitive performance in the Y-maze test, on the other hand, we observed a significant interaction between prenatal environment and postnatal rearing (F_(1, 47)_ = 8.2, *p* = 0.006, Fig. 3F), with post hoc analysis revealing that females that were cross-fostered to mothers of the opposing housing condition showed a significant reduction in time spent in the novel arm compared to females fostered by mothers of the same housing condition as their birth mother. A similar pattern was observed when considering social interaction (significant interaction: F_(1, 47)_ = 5.3, *p* = 0.025, Fig. 3D), however, post hoc multiple comparisons failed to reach statistical significance.

Interactions between the prenatal environment and postnatal care also affected the weight of female offspring at weaning (Supplementary Fig. S3). Specifically, females born to a SIR mother and raised by a GRP foster mother exhibited lower weight at weaning when compared to all other cross-fostering groups. This effect, however, did not maintain statistical significance when the weight of the animals was analysed over time until adulthood (Supplementary Fig. S3). No significant differences were observed when considering the weight of male offspring.

### 3.3 Prenatal and postnatal effects of maternal SIR on offspring gene expression

Lastly, we analysed gene expression in the PFC and AMY of male and female offspring. Target genes were selected based on transcriptomic changes that were previously identified in the SIR model (Scarborough et al., 2021). Hence, our primary aim here was to assess whether such SIR-induced transcriptomic changes were due to prenatal or postnatal factors. In our previous study (Scarborough et al., 2021), we performed next-generation mRNA sequencing (RNAseq) to compare genome-wide transcriptional changes in the AMY of offspring born to GRP or SIR mothers, and then analysed how the observed gene expression changes could translate into different functional readouts using Ingenuity Pathway Analysis (IPA) software (Scarborough et al., 2021). The candidate genes selected in the present study are among the differentially expressed genes uncovered in our previous study, and, more importantly, they all annotate with different behavioral and neuronal functions, such as “learning”, “memory”, “cognition”, “synaptic transmission”, and “neurotransmission”, as identified by IPA (Scarborough et al., 2021). Given the fundamental role of these functions in the behaviors investigated in the present study, our selection of the gene candidates could provide a first insight into the mechanisms underlying the impact of the pre- and postnatal environment on adult behavior.

**Fig. 4:**
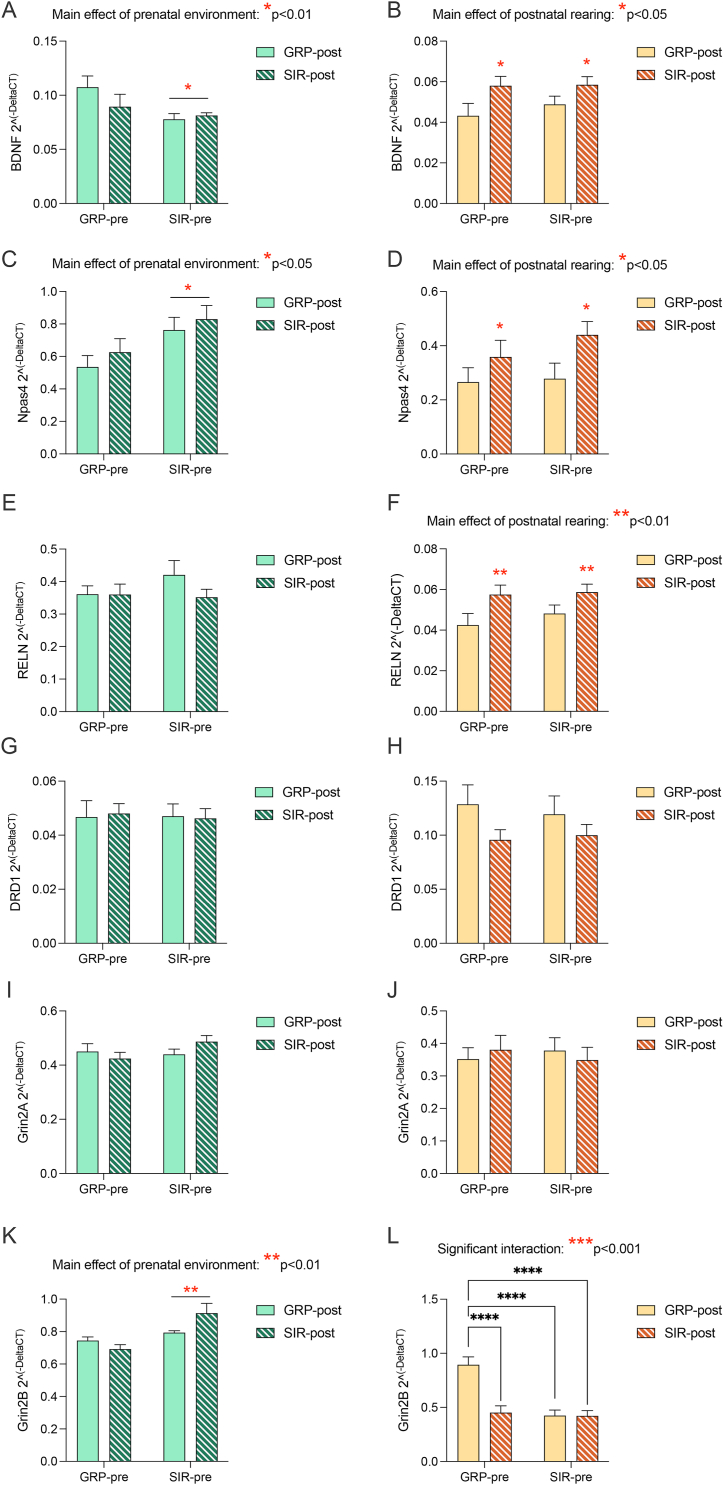
**Effects of pre- and postnatal maternal factors on prefrontal gene expression changes in the offspring.** The bar plots depict mRNA levels in the prefrontal cortex of male (green hues) and female (yellow hues) offspring, respectively. The genes chosen are based on our previous findings in this model, and include (A,B) *Bdnf*, (C, D) *Npas4*, (E, F) *Reln*, (G, H) *Drd1*, (I, J) *Grin2a*, (K, L) *Grin2b* (Post-hoc comparison, female animals: GRP-pre/GRP-post vs GRP-pre/SIR-post ***p < 0.001; GRP-pre/GRP-post vs SIR-pre/GRP-post ***p < 0.001; GRP-pre/GRP-post vs SIR-pre/SIR-post ***p < 0.001). The mRNA levels are reported as 2^^(−deltaCT)^. Significant main effects and interactions uncovered by the 2-way ANOVA are depicted with red asterisks, while significant post-hoc comparisons are depicted with black asterisks. Prenatal conditions are grouped on the x-axis (GRP-pre: offspring born to a GRP dam; SIR-pre: offspring born to a SIR dam), while the colour of the bars indicate the postnatal rearing conditions (GRP-post: offspring raised by a GRP dam; SIR-post: offspring raised by a SIR dam).N = 9–12 male animals per group, N = 11–12 female animals per group.

In the PFC of male offspring, gene expression patterns mirrored the behavioral profile, in that the main driver of the observed changes we uncovered is the prenatal environment. As shown in Fig. 4A, mRNA expression levels of the neurotrophic factor *Bdnf* were downregulated in offspring born to a SIR mother, regardless of whether they were raised by a SIR or GRP foster mother (main effect of prenatal environment: F_(1,39)_ = 5.9, *p* = 0.019). A similar trend was observed when considering the expression levels of the NMDA receptor subunit *Grin2b* and the transcription factor *Npas4.* Specifically, mRNA levels of both these genes were increased in male offspring born to a SIR mother when compared to offspring born to a GRP dam, and these effects were completely irrespective of the foster mother, as demonstrated by the significant main effect of the prenatal environment (Fig. 4K, *Grin2b*: F_(1,39)_ = 10.9, *p* = 0.002; interaction: F_(1,39)_ = 4.3, *p* = 0.046; Fig. 4C, *Npas4*: F_(1,39)_ = 6.6, *p* = 0.013).

**Fig. 5:**
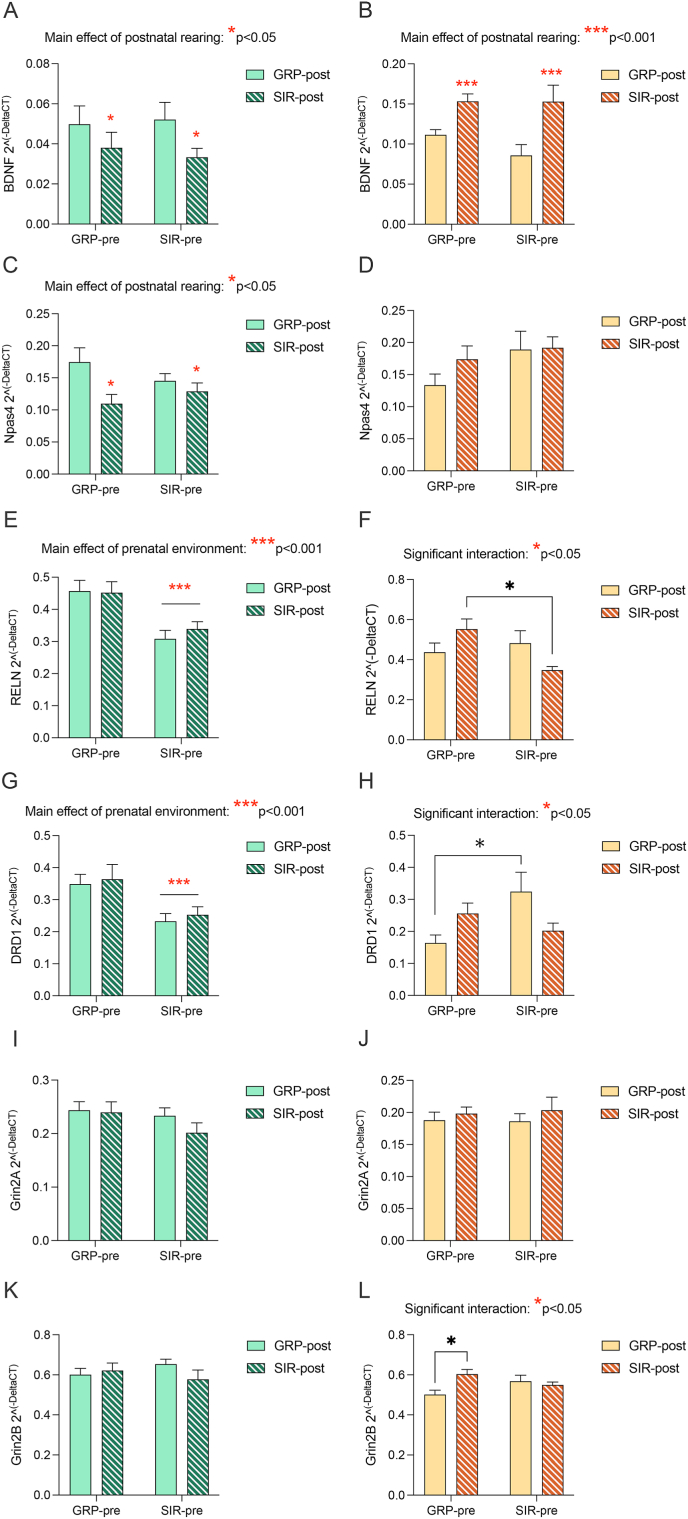
**Effects of pre- and postnatal maternal factors on amygdalar gene expression changes in the offspring.** The bar plots depict mRNA levels in the amygdala of male (green hues) and female (yellow hues) offspring, respectively. The genes chosen are based on our previous findings in this model, and include (A,B) *Bdnf*, (C, D) *Npas4*, (E, F) *Reln* (Post-hoc comparison, female animals: GRP-pre/SIR-post vs SIR-pre/SIR-post *p < 0.05), (G, H) *Drd1* (Post-hoc comparison, female animals: GRP-pre/GRP-post vs SIR-pre/GRP-post *p < 0.05), (I, J) *Grin2a*, (K, L) *Grin2b* (Post-hoc comparison, female animals: GRP-pre/GRP-post vs GRP-pre/SIR-post *p < 0.05). The mRNA levels are reported as 2^^(−deltaCT)^. Significant main effects and interactions uncovered by the 2-way ANOVA are depicted with red asterisks, while significant post-hoc comparisons are depicted with black asterisks. Prenatal conditions are grouped on the x-axis (GRP-pre: offspring born to a GRP dam; SIR-pre: offspring born to a SIR dam), while the colour of the bars indicate the postnatal rearing conditions (GRP-post: offspring raised by a GRP dam; SIR-post: offspring raised by a SIR dam). N = 9–12 male animals per group, N = 11–12 female animals per group.

Interestingly, gene expression patterns slightly differed in the AMY. In detail, *Npas4* and *Bdnf* mRNA expression were selectively reduced in animals raised by a SIR foster mum, irrespective of the birth mother (main effect of postnatal rearing; Fig. 5A, *Bdnf*: F_(1,38)_ = 4.2, p = 0.047; Fig. 5C, *Npas4*: F_(1,38)_ = 6.9, p = 0.012). On the contrary, the expression levels of Drd1 and Reln were reduced in offspring born to a SIR mother, regardless of the postnatal rearing environment (main effect of prenatal factors; Fig. 5G, Drd1: F_(1,38)_ = 13.2, p = 0.0008; Fig. 5E, Reln: F_(1,38)_ = 20.6, p < 0.001). Such results suggest that, while the majority of behavioral and transcriptomic abnormalities seem to be guided, in male offspring, by prenatal factors, some long-lasting gene expression changes may be influenced by the pre- and postnatal environment in a region-specific manner.

When considering the PFC of female offspring, we again observed an opposite pattern compared to males. Indeed, mRNA expression levels of *Bdnf*, *Npas4* and *Reln* were increased in female animals raised by a SIR surrogate mother, independently of their birth mother, as demonstrated by the significant main effect of postnatal rearing (Fig. 4B, *Bdnf*: F_(1,43)_ = 6.9, *p* = 0.011; Fig. 4D, *Npas4*: F_(1,43)_ = 5.1, *p* = 0.028, Fig. 4F, *Reln*: F_(1,43)_ = 7.6, *p* = 0.008). Expression levels of *Grin2b*, on the other hand, were influenced by both pre- and postnatal conditions. Specifically, offspring of SIR dams, regardless of the foster mother, and offspring of GRP dams raised by a SIR surrogate mother, all manifested reduced gene expression levels of NMDA receptor subunit (Fig. 4L, significant main effect of prenatal factors: F_(1,43)_ = 17.9, *p* = 0.0001; interaction: F_(1,43)_ = 13.9, *p* = 0.0006; main effect of postnatal rearing: F_(1,43)_ = 14.2, *p* = 0.0005).

Of note, a main influence of the postnatal rearing environment, and of an interaction between pre-and postnatal factors, was also observed in the AMY. Indeed, mRNA levels of *Bdnf* were increased in female offspring raised by a SIR foster mother, regardless of their birth mother (main effect of postnatal rearing: Fig. 5B, *Bdnf*: F_(1,43)_ = 14.8, p = 0.0004). In addition, expression levels of *Reln*, *Drd1* and *Grin2B* were affected by the interplay between prenatal environment and postnatal care. In detail, expression levels of *Drd1* were increased in female offspring born to a SIR mother and raised by a GRP foster mother (Fig. 5H, interaction: F_(1,43)_ = 6.8, p = 0.011), and the same was true for expression levels of *Grin2b* in GRP offspring raised by a SIR foster mother (Fig. 5L, interaction: F_(1,43)_ = 6.9, p = 0.011). On the other hand, *Reln* expression levels were reduced in SIR offspring raised by a SIR foster mother when compared GRP offspring raised by a SIR foster mother (Fig. 5F, interaction: F_(1,43)_ = 6.6, p = 0.013). These findings point to a complex interplay between the pre and postnatal environment in shaping female development, which is apparent also when considering their behavioral profile.

As a last step, we investigated possible sex-dependent correlations between gene expression levels and behavioral readouts (Supplementary Table S5). Interestingly, we observed, for example, that the gene expression levels of *Bdnf* correlate with behavioral performance of the offspring in a regional and sex-dependent manner. In males, both amygdalar and prefrontal expression of *Bdnf* positively correlate with the relative time that the animals spend in the novel arm of the Y-maze. In females, *Bdnf* expression in the prefrontal cortex positively correlates with the relative time spent exploring the remote object in the temporal order memory test, while the expression levels in the amygdala positively correlate with the relative time spent in the open arm of the elevated plus maze. While such findings merely express a correlation, and should thus be interpreted with caution, they provide support to the hypothesis that the pre- and postnatal environment may affect adult behaviour by altering long-lasting gene expression patterns in different brain areas.

## 4 Discussion

The present study confirms that SIR is effective in inducing a depressive-like state in female dams, which in turn elicits increased anxiety and cognitive deficits in the adult offspring. Here, we went on to show that SIR affects maternal postpartum behavior, and that the behavioral abnormalities in the offspring are impacted in a sex-dependent manner by the prenatal and postnatal environment. Indeed, the cognitive abnormalities and increased anxiety present in male offspring of SIR dams were found to be driven by prenatal factors, as demonstrated by the fact that they were manifest regardless of the surrogate mother that raised these offspring. Alternatively, the behavioral profile of female offspring was found to be shaped by interactions between the prenatal and postnatal milieu. Increased anxiety, for example, was present only in offspring that were reared by a SIR foster mother, regardless of their birth mother, while interactions between pre and postnatal factors shaped their social behavior. Similar sex-dependent effects were observed when considering targeted gene expression in the PFC and AMY. Together, these findings suggest that there may be different time-windows of susceptibility to maternal depression that are dependent on the sex of the offspring (Hodes and Epperson, 2019). Moreover, our data point to different sex-dependent domains of susceptibility, with cognition predominantly impacted in male animals, while social behavior is mainly affected in female animals (Glover and Hill, 2012). Anxiety is affected in both sexes, albeit by different prenatal versus post-natal factors.

Our observations are consistent with existing preclinical and clinical studies (Glover and Hill, 2012). Indeed, when considering sex-dependent time-windows of susceptibility to maternal depression, previous studies have demonstrated that male offspring are more susceptible to the effects of early prenatal stress (Bale et al., 2010). Specifically, prenatal insults have been repeatedly shown to impact males more than females, and the associated risk of developing neurodevelopmental disorders is higher in males as opposed to females (Sandman et al., 2013). This sex-dependent susceptibility to prenatal insults translates into sex biases in the prevalence of various psychiatric disorders, with anxiety and depressive pathologies (that have a post-pubertal onset) more prevalent in women, and neurodevelopmental disorders, such as autism, ADHD and early-onset schizophrenia, more prevalent in men (Sandman et al., 2013; Aleman et al., 2003; Baron-Cohen et al., 2005; Castle and Murray, 1991). Various factors are postulated to play a role in such sex-dependent susceptibility, such as different brain maturation trajectories, which render the male brain more susceptible to insults for a longer period of time, and reduced brain damage compensatory mechanisms in male offspring during fetal development (Geschwind and Galaburda, 1985a, 1985b, 1985c; McGlone, 1978).

The placenta has been demonstrated to have an important role in mediating female resilience and male susceptibility to prenatal insults (Gabory et al., 2013). Indeed, pioneering work conducted by the Bale group demonstrated that placental sex is one of the main drivers of the functional response of the placenta to in-utero perturbations (Bale, 2016). Specifically, they identified that sex differences in the X-linked gene O-linked N-acetylglucosamine transferase (OGT), which plays an important role in transcriptional control and epigenetic programming in the placenta, may underlie increased male susceptibility to neurodevelopmental disorders by affecting how the placenta responds to prenatal insults (Nugent et al., 2018). Other studies point to a role of excess maternal glucocorticoids (GCs) under stressful conditions that could interfere with placental functioning and fetal development. Under physiological conditions, the fetus is shielded by excess maternal GCs by the placental barrier 11β-hydroxysteroid dehydrogenase type 2 (11β-HSD2), which inactivates active forms of GCs (Togher et al., 2014). However, there are sex differences in placental 11β-HSD2 function in response to maternal GCs under stressful conditions, which suggest a better adaptation in females (Zhu et al., 2019). In addition, male placentas have shown worse adaptation to undernutrition associated with lower efficiency, oxidative disbalance and reduced vascularization and glucocorticoid barrier (Phuthong et al., 2020). While our study was not designed to investigate specific factors acting in-utero, our results align with these findings in that the behavioral alterations and gene expression changes observed in male offspring were largely driven by the prenatal environment, and thus by stress-dependent effects that manifest in-utero. Further studies are warranted to investigate possible sex-dependent placental molecular signatures and functions in the SIR model.

In contrast, our results suggest that female offspring may be more susceptible to alterations in maternal care when compared to their male littermates, as adult behavioral alterations were either present in offspring reared by a SIR mother, regardless of their prenatal background, or the result of complex interactions between the pre-and post-natal environment. The effects of altered maternal care on female offspring have been observed in a variety of different studies. For example, previous studies using similar cross-fostering designs in mouse models of maternal immune activation (MIA) found that the sex-dependent development of abnormal fear expression was driven by postnatal but not prenatal maternal factors (Schwendener et al., 2009). In these MIA models, exposure to viral-like immune activation during late gestation led to alterations in post-partum maternal behavior, which in turn was found to mediate increased fear expression in female but not male offspring (Schwendener et al., 2009). Hence, consistent with our findings, these previous results suggested that female offspring may be more sensitive than males in terms of the negative effects induced by changes in postpartum maternal care, at least when considering fear-related behaviors. Interestingly, the same MIA models further provided evidence that prenatal but not postnatal maternal factors mediate MIA-induced cognitive deficits in the offspring (Richetto et al., 2013), akin to what we have observed in male offspring born to SIR-exposed mothers.

Sex-dependent effects were also observed when considering gene expression. Male offspring exhibit transcriptional changes in the PFC, and to some extent also in the AMY, which are mainly driven by the prenatal environment, as when considering their behavioral profile. Females, on the other hand, present transcriptional changes in both brain regions that are either driven by postnatal factors, or that result from the interplay between pre and postnatal environment, mirroring the complex picture that emerges when analyzing their behavioral performance. Such overlap between sex-dependent behavioral and transcriptional alterations provides further support to the hypothesis that pre and postnatal factors may have an effect on behavioral profiles by impacting the transcription of the candidate genes investigated in the present study. While our experiments are far from proving any mechanistic or causative link between gene expression and behavior, the selected gene candidates are well known to be involved in regulating the behaviors that are altered in SIR offspring. The neuron specific immediate-early gene *Npas4,* for example, has been shown to have a role in memory function, converting experience induced neuronal firing into downstream changes (Sun and Lin, 2016). Likewise, the neurotrophic factor *Bdnf* has long been associated with synaptic plasticity and cognitive function (Lu et al., 2014), and has also been shown to play a role in anxiety-related behaviors alongside *Grin2b* (Hashimoto, 2007; Yan et al., 2017). Interestingly, here we show that the gene expression levels of *Bdnf* correlate with some aspects of cognitive performance and anxiety behavior in a regional and sex-dependent manner. Together with our previous findings, which annotated transcriptional dysregulation of these genes with specific behavioral and neuronal functions (Scarborough et al., 2021), our present data thus point to possible mechanistic targets of pre and postnatal factors that warrant further subsequent investigations to probe their causative role in mediating the effects of SIR on offspring behavior.

When considering maternal behavior, it is well known that mother–infant interactions are reciprocal (Fleming et al., 1999; Walker et al., 2004). Specifically, the rearing mother's care influences the physiological and neurobiobehavioral development of the offspring, while in turn the offspring's affiliative behavior and demands critically determine how competent maternal care will be. In our experimental design, each surrogate mother concomitantly raised pups of both prenatal treatment conditions. This ensured that each surrogate mother was exposed to similar affiliative behaviors and demands originating from the pups. Therefore, we can rule out the possibility that the changes in maternal behavior observed in SIR surrogate mothers may result from possible differences in affiliative behavior and/or demands between offspring born to SIR and control mothers. Rather, the identified alterations in maternal care are likely to be accounted for by the SIR-associated processes taking place in gestation. Given the subtle nature of the changes in maternal behavior that we observed, however, we cannot rule out that other postnatal maternal factors, such as postpartum metabolic alterations induced by SIR, may also play a role in affecting the post-partum milieu. Metabolic effects of SIR seem plausible given the effect of such housing regime on the weight gain of dams and offspring seen here and in our previous study (Scarborough et al., 2021) and thus warrant future investigations, that, however, go beyond the scope of the present investigation.

Our findings highlighting also that the timing-dependent and sexually dimorphic effects of SIR on cognitive and affective development in the offspring are in line with numerous clinical data. For example, research focusing on cognitive development in the offspring has found that the prenatal, not the postpartum, period is the most sensitive period of development, in which depressive symptoms in the mother have the strongest effect on cognitive functioning in offspring when experienced during the antenatal period (Evans et al., 2012). This finding adds to the growing data which implicate depression in the prenatal period as the most important period for a range of aspects of child development (Deave et al., 2008; Hay et al., 2010; O'Connor et al., 2003). However, maternal depression in the postpartum period has also been shown to have an effect on the cognitive development of the offspring, an effect emerging more substantially in with male offspring (Hay et al., 2008). In addition, exposure to postpartum depression has also been associated with mood disorders, including an increased risk for depression and anxiety (Halligan et al., 2007; Murray et al., 2011). Consistent with our data, female offspring were found to be more susceptible than males in terms of developing mood disorders after exposure to postpartum depression (Halligan et al., 2007).

Our study presents with some limitations and possible confounders. Firstly, while we specifically chose to induce a depressive-like phenotype in the dams by exposing them to social isolation rearing, we acknowledge that such experimental paradigm is associated with a more severe form of stress as compared to conditions in which social isolation is induced in adulthood, as the animals are deprived of important social stimuli already in adolescence. Post-weaning isolation leads to a depressive-like state that arises before mating and pregnancy (Scarborough et al., 2021), which in turn more readily mimics the clinical scenario in which previous episodes of depression and early life stressors, both important risk factors for depression in pregnancy (Biaggi et al., 2016; Plant et al., 2013, 2018), are present before gestation. While this responds to the specific aims of our study, we cannot exclude that such early social deprivation may represent an additional confounder whose effect cannot be disentangled from that of the stress induced by social deprivation in adulthood. Further studies that assess the effects of shorter isolation periods later in life would be needed to rule out such confounders. Moreover, our study is limited in the fact that it was not designed to assess the molecular mechanisms underlying the behavioral observations. Despite this, however, our data highlight the complex interaction between antenatal and postnatal maternal factors, and the sex of the offspring, in determining the impact of maternal depression on the offsprings’ behavioral profile. Our findings expand previous work by implementing a novel and clinically-relevant model of maternal depression, and underscore the importance of including offspring sex as a fundamental variable in antepartum and postpartum depression research. Future mechanistic studies in this model may help advance knowledge regarding the complex underpinnings of such interactions.

## CRediT authorship contribution statement

**Joseph Scarborough:** Conceptualization, Data curation, Formal analysis, Funding acquisition, Investigation, Writing – review & editing, Methodology, Validation. **Monica Iachizzi:** Data curation, Investigation, Validation. **Sina M. Schalbetter:** Data curation, Investigation, Validation. **Flavia S. Müller:** Data curation, Investigation. **Ulrike Weber-Stadlbauer:** Methodology, Supervision. **Juliet Richetto:** Conceptualization, Formal analysis, Funding acquisition, Methodology, Project administration, Resources, Supervision, Writing – original draft, Writing – review & editing.

## Declaration of competing interest

None

## Data Availability

Data will be made available on request.
