# Supplementary material for: Prenatal and postnatal influences on behavioral development in a mouse model of preconceptional stress
Source: Neurobiol Stress. 2024 Feb 3;29:100614. doi: 10.1016/j.ynstr.2024.100614 (PMC10865047; doi:10.1016/j.ynstr.2024.100614)
Supplement: Multimedia component 1 [file mmc1.docx]

**Prenatal and postnatal influences on behavioral development in a mouse model of preconceptional stress**

Joseph Scarborough^1^, Monica iachizzi^1^, Sina Schalbetter^1^, Flavia Müller^1^, Ulrike Weber-Stadlbauer^1,2^, Juliet Richetto^1,2^*

^1^Institute of Pharmacology and Toxicology, University of Zurich-Vetsuisse, Zurich, Switzerland.

^2^Neuroscience Center Zurich, University of Zurich and ETH Zurich, Zurich, Switzerland.

***Correspondence:**

Dr. Juliet Richetto, Ph.D., Institute of Pharmacology and Toxicology, University of Zurich-Vetsuisse, Winterthurerstrasse 260, 8057 Zurich, Switzerland.

E-mail: [juliet.richetto@uzh.ch](mailto:juliet.richetto@uzh.ch); Tel.: +41 44 635 87 77; Fax.: +41 44 635 89 10.

1. **Supplementary methods**
   1. **Experimental allocation of animals**

| **Maternal Housing** | **Animals** | **Testing and Mating** | **Dams monitored for Maternal Behavior** | **Cross-fostered Litters** | **Pups included in Behavioral Testing** | **Pups included in Postmortem Analysis** |
| --- | --- | --- | --- | --- | --- | --- |
| SIR | 20F | 20 (SI, OF) | 19 | 11 | 9M, 13F SIR-GRP | 9M, 12F SIR-GRP |
|  |  |  |  |  | 12M, 12F SIR-SIR | 12M, 11F SIR-SIR |
| GRP | 16F | 16 (SI, OF) | 13 | 11 | 11M, 12F GRP-GRP | 11M, 12F GRP-GRP |
|  |  |  |  |  | 11M, 14F GRP-SIR | 11M, 12F GRP-SIR |

**Supplementary Table S1.** Number and allocation of socially isolated (SIR) and group-housed (GRP) female animals. The table also specifies the final number of cross-fostered litters in each group, and the final number of offspring (the same offspring were used for behavior and postmortem analysis – unfortunately, 4 females were excluded from the postmortem analysis due to loss at sacrifice). The number of dams and litters included in each group was based on previous studies conducted in our laboratory based on other prenatal manipulation models.

**1.2 Cross-fostering of the offspring**

**Supplementary Figure S1.** Experimental design for the neonatal cross-fostering procedure, which resulted in four different groups depending on the birth (prenatal) and foster mother (postnatal): 1) GRP-GRP 2) GRP-SIR 3) SIR-GRP 4) SIR-SIR; with isolated mother mice and her pups in green, group housed mice in beige.

**1.3 Maternal behaviour**

Detailed description of each behavioral category is as follows:

Licking/grooming: The mother is licking and/or grooming the pups, clearly recognizable by the movement of her head and/or paws.

Self-licking/-grooming: Self-directed licking and/or grooming behavior when the mother is in the nest; self-directed licking and/or grooming behavior occurring outside the nest would be scored as “off-nest” (see below).

Eating/drinking: The mother is eating and drinking from food magazine and water bottles, respectively.

Nursing: Feeding the pups; this comprises all categories of nursing, including kyphosis (nursing the pups in a dorsal upright position, with rigid fore- and/or hindlimbs) and prone nursing (nursing the pups while lying flat on the top of the pups, with little or no limb support).

Nest-building: The mother is moving sawdust around the nest or is preparing a new nest; in addition, moving pups from one nest to another is included in this category.

Off nest: This is scored when the mother is somewhere in the cage without any contact to the pups; this category does not include the categories of “self-licking/-grooming”, “eating/drinking” or “nest-building”.

**1.4 Dam and offspring behavioral testing**

The behavioral testing was conducted as follows:

*Light Dark box test*

The light dark box test is analysed using 4 identical Multi Conditioning boxes (Multi Conditioning System, Germany) each with a dark (1 Lux) and a bright (100 Lux) chamber, separated from each other by a dark plexiglass wall, within which there is an electrically controlled door. Each mouse is placed in the dark compartment. After 5 seconds the door automatically opens, allowing access to both the dark and bright compartment for 5 minutes. The measurements collected from this test include the latency to move into the bright compartment from the dark compartment and the total time spent in each compartment.

*Elevated Plus maze*

The elevated plus maze tests served as tests for innate anxiety-like behaviour. The apparatus was made of Plexiglas painted in grey and consisted of 4 equally spaced arms (5 x 30 cm) radiating from a square centre (5 × 5 cm). One pair of opposing arms was enclosed with opaque walls (height: 15 cm) except for the side adjoining the central square (CZ). The remaining two arms were exposed with a parameter border (height: 3 mm) along the outer edges. The maze was elevated 70 cm above floor level and positioned in a testing room with diffused lighting of 30 lux on the exposed arms. A digital camera was mounted above the plus maze, captured images at a rate of 5 Hz and transmitted them to a PC running the Ethovision (Noldus Technology, Wageningen, The Netherlands) tracking system. A test session began by placing the animal into the CZ with it facing one of the closed arms. It was then left to explore freely for 5 min before being returned to the home cage. After each trial, the apparatus was cleansed with water and dried before a new trial began. The relative (percent) time spent in the open arms and relative open arm entries during the entire 5- min test period were analyzed in order to index anxiety-related behavior. The percent time spent in the open arms was calculated using the formula [(time spent in the open arms) / (time spent in all arms) × 100]. In addition, total distance moved in the entire maze was analyzed in order to assess general locomotor activity

*Social interaction test*

Social interaction was assessed by analyzing the relative exploration time between an unfamiliar congenic mouse and an inanimate dummy object using methods established before^1^. The test apparatus was made of Plexiglas and consisted of three identical arms (50 cm × 9 cm; length × width) surrounded by 10-cm high Plexiglas walls. The three arms radiated from a central triangle (8 cm on each side) and spaced 120° from each other. Two out of the three arms contained a rectangular wire grid cage (13 cm × 8 cm × 10 cm, length × width × height; bars horizontally and vertically spaced 9 mm apart). The third arm did not contain a metal wire cage and served as the start zone (see below).

During the test phase, one metal wire cage contained an unfamiliar C57BL6/N mouse of the same sex and age, whereas the other wire cage contained an inanimate dummy object. The latter was a black scrunchie made of velvet material. The allocation of the unfamiliar live mouse and inanimate dummy object to the two wire cages was counterbalanced across experimental groups. To start a test trial, the test mouse was gently placed in the start arm and allowed to explore freely for 5 min. Behavioral observations were made by an experimenter blind to the experimental conditions, and social interaction was defined as nose contact within a 2-cm interaction zone. The relative time spent with the live mouse was calculated by the formula ([time spent with the mouse]/[time spent with the inanimate object + time spent with the mouse]) × 100 and used to compare the relative exploration time between the unfamiliar mouse and the inanimate dummy object. The total distance moved during the test was also measured to analyze general exploratory activity. This was achieved by a digital camera mounted above the apparatus, which provided images at a rate of 5 Hz that were transmitted to a PC running the EthoVision tracking system (Noldus, Wageningen, The Netherlands).

*Open-field test*

The open-field test was conducted in 4 identical open-field arenas (40 × 40 × 35-cm high) made of white plastic as similarly described in^2,3^. They were located in a testing room under dim diffused lighting (approximately 35 lux as measured in the center of the arenas). A digital camera was mounted directly above the 4 arenas. Images were captured at a rate of 5 Hz and transmitted to a PC running the Ethovision (Noldus, Wageningen, The Netherlands) tracking system to record locomotor activity indexed by the distance moved in the entire open field arena. The animals were placed into the center of the open field arena and allowed to explore freely for 30 min. At the end of this time period, the animals were removed from the apparatus and returned to their home cage.

*Y-maze-Novel arm recognition*

Y-maze-novel arm recognition was assessed by analysing the relative exploration time between a novel arm and a familiar arms of the y-maze. The test apparatus was made of Plexiglas and consisted of three identical arms (50 cm × 9 cm; length × width) surrounded by 10-cm high Plexiglas walls. The three arms radiated from a central triangle (8 cm on each side) and spaced 120° from each other.

During the first phase the mice are placed in the start arm, with one of the other arms blocked entrance using opaque plexiglass divider. The mice are allowed to freely explore two arms of the y-maze for 5 minutes before being removed. After the mice are removed, they are placed back into the transport box for 1 minute, while the plexiglass divider is removed. To start the test trial the mice are gently placed again into the start arm and are left to freely explore the entire y-maze for 5 minutes. The relative time spent in the novel arm was calculated by the formula ([time spent in the novel arm]/[time spent in familiar arm 1 + time spent in familiar arm 2+ time spent in unfamiliar arm]) × 100 and used to compare the relative time spent exploring the novel arm and the familiar arms. The total distance moved during the test was also measured to analyse general exploratory activity. This was achieved by a digital camera mounted above the apparatus, which provided images at a rate of 5 Hz that were transmitted to a PC running the EthoVision tracking system (Noldus, Wageningen, The Netherlands).

*Temporal order memory test*

The temporal order memory test was conducted in 4 identical open-field arenas (40 × 40 × 35-cm high) made of white plastic as similarly described in^2,3^. They were located in a testing room under dim diffused lighting (approximately 35 lux as measured in the center of the arenas). A digital camera was mounted directly above the 4 arenas. Images were captured at a rate of 5 Hz and transmitted to a PC running the Ethovision (Noldus, Wageningen, The Netherlands) tracking system to record locomotor activity indexed by the distance moved in the entire open field arena.

During the first phase the animals were gently placed in the centre of the empty open-field arena and allowed to freely explore the arena for 10 minutes before being removed. After the first phase the animals are placed in a holding room for 30 minutes before the start of the next phase. In the second phase two identical objects are placed in the open-field arena in opposing corners approx. 5cm from the walls. The mice are again gently placed into the centre of the open-field arenas and allowed to freely interact with the objects contained within for 10 minutes, the mice are again removed and placed in the holding room for 30 minutes before the start of the next phase. Before the start of the 3^rd^ phase the objects are replaced with a new set of identical objects place in the same position as the first objects. The mice are placed again into the open-field arena and allowed to explore the object. The mice are again removed from the open-field after 10 minutes and placed in the holding room for 30 minutes. For the final test phase one of the first and one of the second presented objects are placed in the open-field arena again in the same position as the previous objects, counterbalanced. To assess the recognition of the temporally remote object the percentage time spent interacting with the remote object during the test phase was calculated using the formula ([time spent interacting with the remote object]/[time spent interacting with the remote object + time spent interacting with the recent object]) × 100, this can be used to determine the preference for the remote objected compared to the more recent object.

**1.2.7 RNA extraction and Quantitative Real-Time PCR Analyses**

Following brain dissection, mRNA was extracted from the frozen brain punches with the SPLIT kit (Lexogen, Germany) according to manufacturer’s recommendations. RNA was analyzed by TaqMan qRT-PCR instrument (CFX384 real-time system, Bio-Rad Laboratories) using the iScript one-step RT-PCR kit for probes (Bio-Rad Laboratories). The samples were run in 384-well formats in triplicates as multiplexed reactions with a normalizing internal control (36B4). We choose 36B4 as internal standard for gene expression analyses since its expression was not affected by the prenatal treatments.

Thermal cycling was initiated with an incubation at 50°C for 10 min (RNA retrotranscription) and then at 95°C for 5 min (TaqMan polymerase activation). After this initial step, 39 cycles of PCR were performed. Each PCR cycle consisted of heating the samples at 95°C for 10 s to enable the melting process and then for 30 s at 60°C for the annealing and extension reaction. Relative target gene expression was calculated according to the 2(-Delta Delta C(T)) method^4^. Custom-designed probe and primer sequences, or product codes, used for the various genes of interest and reference gene (36B4) are summarized below and were purchased from Eurofins Genomics GmbH (Germany) and from Thermo Fisher Scientific (Germany).

| **Gene** | **Forward primer** | **Reverse primer** | **Probe** |
| --- | --- | --- | --- |
| Bdnf | 5’-AAGTCTGCATTACATTCCTCGA-3’ | 5’-GTTTTCTGAAAGAGGGACAGTTTAT-3’ | 5’-TGTGGTTTGTTGCCGTTGCCAAG-3’ |
| Reln | 5’-GGGTATAATCGGAATGTCTGGG-3’ | 5’-AGTAGAAAACTCCAAGCTGACG-3’ | 5’-TGTTGAAGGGAGAACGCGCAGC-3’ |
| Npas4 | 5’-GTCCTAATCTACCTGGGCTTTG-3’ | 5’-TCTCCACTTTCAGCCAACAG-3’ | 5’-ATGGTATGGACTGCTACACCCCGA-3’ |
| 36b4 | 5’-AGATGCAGCAGATCCGCAT-3’ | 5’-GTTCTTGCCCATCAGCACC-3’ | 5’-CGCTCCGAGGGAAGGCCG-3’ |
| **Gene** | **Product code** | | |
| Grin2a | Mm00433802_m1 | | |
| Grin2b | Mm00433820_m1 | | |
| Drd1 | Mm02620146_s1 | | |

**Supplementary Table S2.** Custom-designed probe and primer sequence (Eurofins), or product codes (Thermo Fisher Scientific), of the genes investigated with quantitative Real-Time PCR analyses.

**2. Supplementary results**

**2.1 Effects of SIR on weight gain in female animals before and during pregnancy**

**A B C**

**Supplementary Figure S2: Effects of SIR on weight gain in female animals before and during pregnancy.** (A) The line plots depict the weight gain from weaning to adulthood of GRP (in yellow) and SIR (in green) females, respectively. GRP vs SIR on PND70, PND77 and PND84: *p<0.05 (B, C) The bar plots depict the weight of SIR and GRP females at PND84, before mating, and at the beginning of the first and third week of pregnancy, respectively. GRP vs SIR on PND84: *p<0.05 N = 16-20 females per group.

As depicted in Supplementary Figure S2, SIR led to increased weight gain in female mice, an observation that is supported by the significant time x weight interaction (*F*_(9,306)_ = 3.904, *p* < 0.0001). This is also confirmed by a representative comparison at PND84, depicted by the bar plots in B (t_(34)_ = 0.0197). The bar plots in C depict the weight gain at the beginning of the first and third week of pregnancy, respectively. No significant difference was observed between the two groups.

**2.2 Effects of SIR on litter size**

** A**

**Supplementary Figure S3: Effects of SIR on litter size.** (A) The bar plots depict the average number of pups per litter in GRP (in yellow) and SIR (in green) females, respectively. N = 11 litters per group.

As depicted in Supplementary Figure S3, we did not observe any significant difference in terms of litter size between the two groups. This is consistent with our previous findings in the SIR model^5^.

**2.3 Effects of SIR on weight gain in female and male offspring**

**A B**

**C D**

**Supplementary Figure S3: Effects of SIR on weight gain in female and male offspring.** (A,C) The bar plots depict the weight of GRP-pre/GRP-post, GRP-pre/SIR-post, SIR-pre/GRP-post and SIR-pre/SIR-post female (yellow hues) and male (green hues) offspring at PND21, at weaning, respectively. (B,D) The line plots depict the weight gain from weaning to adulthood of GRP-pre/GRP-post, GRP-pre/SIR-post, SIR-pre/GRP-post and SIR-pre/SIR-post female (yellow and pink hues) and male (green and brown hues) offspring, respectively. N = 9-14 animals per group. GRP-pre/GRP-post vs SIR-pre/GRP-post **p* < 0.05; GRP-pre/SIR-post vs SIR-pre/GRP-post **p* < 0.05; SIR-pre/GRP-post vs SIR-pre/SIR-post ***p* < 0.01.

As depicted in Supplementary Figure S3, the pre- and postnatal environment had an effect on female offspring weight at weaning, as demonstrated by the main effect of prenatal environment (*F*_(1,47)_ = 7.74, *p* < 0.0001) and the significant interaction between prenatal environment and postnatal rearing (*F*_(1,47)_ = 7.78, *p* < 0.0001). Specifically, females born to a SIR mother and raised by a GRP foster mother exhibited lower weight at weaning when compared to all other cross-fostering groups. This effect, however, did not maintain statistical significance when the weight of the animals was analyzed over time until adulthood (line plot). No significant differences were observed in male offspring.

**2.4 Summary of behavioral and gene expression statistical analysis in males**

|  | | **2-way ANOVA (*p* values)** | | |
| --- | --- | --- | --- | --- |
| **Measure** | **Readout** | **Prenatal Environment** | **Postnatal Care** | **Interaction** |
| **Male behavior** | Elevated plus maze - **% Time in the open arm** | *0.0232* | 0.1943 | 0.5703 |
|  | Social interaction test - **% Time live mouse** | 0.2493 | 0.3264 | 0.5946 |
|  | Y-maze test - **% Time novel arm** | *0.0084* | 0.9436 | 0.6144 |
|  | TOMT - **% Time remote object** | *0.0302* | 0.2531 | 0.0753 |
| **Male gene expression –**  **PFC** | *Bdnf* | *0.0194* | 0.3579 | 0.1735 |
|  | *Npas4* | *0.0137* | 0.3530 | 0.8826 |
|  | *Reln* | 0.4411 | 0.2974 | 0.3087 |
|  | *Drd1* | 0.8704 | 0.9582 | 0.8193 |
|  | *Grin2b* | *0.0020* | 0.4123 | *0.0426* |
|  | *Grin2a* | 0.2887 | 0.6723 | 0.1356 |
| **Male gene expression –**  **AMY** | *Bdnf* | 0.8730 | *0.0474* | 0.6393 |
|  | *Npas4* | 0.7478 | *0.0120* | 0.1259 |
|  | *Reln* | *<0.0001* | 0.6539 | 0.5364 |
|  | *Drd1* | *0.0008* | 0.5719 | 0.9343 |
|  | *Grin2b* | 0.9121 | 0.4788 | 0.2160 |
|  | *Grin2a* | 0.1844 | 0.3229 | 0.4378 |

**Supplementary Table S3.** Summary of 2-way ANOVA analysis of behavioral readouts and gene expression levels in male animals. The table reports the *p* values of the main effects of prenatal environment and postnatal care and their interaction obtained in the 2-way ANOVA analysis. In red *p* values that reach statistical significance.

**2.5 Summary of behavioral and gene expression statistical analysis in females**

|  | | **2-way ANOVA (*p* values)** | | |
| --- | --- | --- | --- | --- |
| **Measure** | **Readout** | **Prenatal Environment** | **Postnatal Care** | **Interaction** |
| **Female behavior** | Elevated plus maze - **% Time in the open arm** | 0.6526 | *0.0078* | 0.4876 |
|  | Social interaction test - **% Time live mouse** | 0.3175 | 0.4665 | *0.0255* |
|  | Y-maze test - **% Time novel arm** | 0.3377 | 0.4113 | *0.0062* |
|  | TOMT - **% Time remote object** | 0.2799 | 0.6411 | 0.5050 |
| **Female gene expression –**  **PFC** | *Bdnf* | 0.5137 | *0.0115* | 0.5817 |
|  | *Npas4* | 0.4076 | *0.0285* | 0.5446 |
|  | *Reln* | 0.4572 | *0.0082* | 0.6287 |
|  | *Drd1* | 0.8570 | 0.0647 | 0.6293 |
|  | *Grin2b* | *0.0001* | *0.0005* | *0.0006* |
|  | *Grin2a* | 0.9484 | 0.9941 | 0.4751 |
| **Female gene expression –**  **AMY** | *Bdnf* | 0.3615 | *0.0004* | 0.3733 |
|  | *Npas4* | 0.1006 | 0.3289 | 0.3944 |
|  | *Reln* | 0.1071 | 0.8467 | *0.0131* |
|  | *Drd1* | 0.1989 | 0.7159 | *0.0119* |
|  | *Grin2b* | 0.7768 | 0.0740 | *0.0115* |
|  | *Grin2a* | 0.8975 | 0.3439 | 0.8186 |

**Supplementary Table S4.** Summary of 2-way ANOVA analysis of behavioral readouts and gene expression levels in female animals. The table reports the *p* values of the main effects of prenatal environment and postnatal care and their interaction obtained in the 2-way ANOVA analysis. In red *p* values that reach statistical significance.

**2.6 Pearson correlation analysis between behavioral and gene expression data**

| **Pearson Correlation** | | **Behavioral readout** | | | |
| --- | --- | --- | --- | --- | --- |
| **Brain Region** | **Gene** | **% Time in the open arm** | **% Time novel arm** | **% Time remote object** | **% Time live mouse** |
| **Male PFC** | *Bdnf* | r = 0.1198 | r = 0.4182 | r = 0.2582 | r = 0.08724 |
|  |  | *p* = 0.4615 | *p* = *0.0072* | *p* = 0.1077 | *p* = 0.5924 |
|  | *Npas4* | r = 0.03654 | r = -0.2169 | r = -0.05435 | r = 0.1660 |
|  |  | *p* = 0.8252 | *p* = 0.1847 | *p* = 0.7424 | *p* = 0.3124 |
|  | *Reln* | r = 0.1091 | r = -0.1081 | r = 0.05173 | r = -0.2935 |
|  |  | *p* = 0.4915 | *p* = 0.4955 | *p* = 0.7449 | *p* = 0.0592 |
|  | *Drd1* | r = 0.2221 | r = 0.2154 | r = 0.02093 | r = 0.2095 |
|  |  | *p* = 0.1575 | *p* = 0.1707 | *p* = 0.8953 | *p* = 0.1831 |
|  | *Grin2b* | r = 0.2995 | r = -0.05684 | r = -0.06077 | r = -0.1611 |
|  |  | *p* = 0.0540 | *p* = 0.7207 | *p* = 0.7022 | *p* = 0.3080 |
|  | *Grin2a* | r = 0.2061 | r = 0.04717 | r = 0.1532 | r = -0.07543 |
|  |  | *p* = 0.1904 | *p* = 0.7667 | *p* = 0.3328 | *p* = 0.6349 |
| **Male AMY** | *Bdnf* | r = -0.02575 | r = 0.3321 | r = 0.2153 | r = 0.06560 |
|  |  | *p* = 0.8764 | *p* = *0.0389* | *p* = 0.1881 | *p* = 0.6915 |
|  | *Npas4* | r = -0.05794 | r = 0.2558 | r = 0.2515 | r = 0.3193 |
|  |  | *p* = 0.7225 | *p* = 0.1111 | *p* = 0.1175 | *p* = *0.0446* |
|  | *Reln* | r = -0.1839 | r = -0.04535 | r = 0.1683 | r = 0.1786 |
|  |  | *p* = 0.2496 | *p* = 0.7783 | *p* = 0.2929 | *p* = 0.2639 |
|  | *Drd1* | r = -0.2271 | r = -0.03844 | r = 0.1836 | r = 0.2084 |
|  |  | *p* = 0.1704 | *p* = 0.8188 | *p* = 0.2699 | *p* = 0.2092 |
|  | *Grin2b* | r = -0.2666 | r = -0.09839 | r = 0.1901 | r = 0.1140 |
|  |  | *p* = 0.0964 | *p* = 0.5458 | *p* = 0.2401 | *p* = 0.4837 |
|  | *Grin2a* | r = -0.3434 | r = -0.05752 | r = 0.04129 | r = 0.1358 |
|  |  | *p* = *0.0323* | *p* = 0.7280 | *p* = 0.8029 | *p* = 0.4097 |
| **Female PFC** | *Bdnf* | r = -0.04054 | r = 0.1371 | r = 0.3625 | r = -0.06190 |
|  |  | *p* = 0.7939 | *p* = 0.3693 | *p = 0.0144* | *p* = 0.6863 |
|  | *Npas4* | r = -0.2262 | r = -0.03831 | r = 0.03543 | r = 0.02362 |
|  |  | *p* = 0.1399 | *p* = 0.8005 | *p* = 0.8152 | *p* = 0.8762 |
|  | *Reln* | r = -0.1049 | r = 0.01392 | r = 0.2357 | r = -0.03211 |
|  |  | *p* = 0.4979 | *p* = 0.9268 | *p* = 0.1148 | *p* = 0.8322 |
|  | *Drd1* | r = 0.2362 | r = 0.08637 | r = 0.1288 | r = 0.06434 |
|  |  | *p* = 0.1182 | *p* = 0.5637 | *p* = 0.3882 | *p* = 0.6674 |
|  | *Grin2b* | r = -0.1036 | r = -0.2046 | r = 0.2731 | r = 0.1779 |
|  |  | *p* = 0.4933 | *p* = 0.1631 | *p* = 0.0604 | *p* = 0.2263 |
|  | *Grin2a* | r = -0.02337 | r = -0.1715 | r = 0.2598 | r = 0.04316 |
|  |  | *p* = 0.8775 | *p* = 0.2437 | *p* = 0.0745 | *p* = 0.7709 |
| **Female AMY** | *Bdnf* | r = -0.3483 | r = 0.05035 | r = -0.05585 | r = 0.05658 |
|  |  | *p* = *0.0177* | *p* = 0.7397 | *p* = 0.7124 | *p* = 0.7088 |
|  | *Npas4* | r = 0.1346 | r = 0.08514 | r = -0.1565 | r = -0.01456 |
|  |  | *p* = 0.3896 | *p* = 0.5737 | *p* = 0.2990 | *p* = 0.9235 |
|  | *Reln* | r = -0.01543 | r = -0.1525 | r = -0.01494 | r = 0.1071 |
|  |  | *p* = 0.9218 | *p* = 0.3117 | *p* = 0.9215 | *p* = 0.4786 |
|  | *Drd1* | r = 0.3497 | r = 0.03352 | r = 0.08803 | r = -0.02696 |
|  |  | *p* = *0.0232* | *p* = 0.8290 | *p* = 0.5699 | *p* = 0.8621 |
|  | *Grin2b* | r = -0.1723 | r = 0.01171 | r = -0.08384 | r = 0.01789 |
|  |  | *p* = 0.2522 | *p* = 0.9364 | *p* = 0.5668 | *p* = 0.9029 |
|  | *Grin2a* | r = -0.1597 | r = 0.02336 | r = -0.03913 | r = 0.1551 |
|  |  | *p* = 0.2890 | *p* = 0.8734 | *p* = 0.7895 | *p* = 0.2872 |

**Supplementary Table S5.** Pearson correlation analysis of behavioral and gene expression data in male and female animals. The table reports the Pearson’s correlation coefficient (r) and *p* values of the correlations. In red *p* values that reach statistical significance.

As summarized in Supplementary Table S5, we observed some sex-dependent correlations between regional gene expression and behavioral performance of the animals in the behavioral tests included in the study. While such findings merely express a correlation, and should thus be interpreted with caution, they provide support to the hypothesis that the pre- and postnatal environment may affect adult behaviour by altering long-lasting gene expression patterns in different brain areas.

**Bibliography**

1 Weber-Stadlbauer, U., Richetto, J., Labouesse, M. A., Bohacek, J., Mansuy, I. M. & Meyer, U. Transgenerational transmission and modification of pathological traits induced by prenatal immune activation. *Mol Psychiatry* **22**, 102-112, doi:10.1038/mp.2016.41 (2017).

2 Meyer, U., Nyffeler, M., Schwendener, S., Knuesel, I., Yee, B. K. & Feldon, J. Relative prenatal and postnatal maternal contributions to schizophrenia-related neurochemical dysfunction after in utero immune challenge. *Neuropsychopharmacology* **33**, 441-456, doi:10.1038/sj.npp.1301413 (2008).

3 Meyer, U., Nyffeler, M., Yee, B. K., Knuesel, I. & Feldon, J. Adult brain and behavioral pathological markers of prenatal immune challenge during early/middle and late fetal development in mice. *Brain Behav Immun* **22**, 469-486, doi:10.1016/j.bbi.2007.09.012 (2008).

4 Livak, K. J. & Schmittgen, T. D. Analysis of relative gene expression data using real-time quantitative PCR and the 2(-Delta Delta C(T)) Method. *Methods* **25**, 402-408, doi:10.1006/meth.2001.1262 (2001).

5 Scarborough, J., Mueller, F. S., Weber-Stadlbauer, U., Mattei, D., Opitz, L., Cattaneo, A. & Richetto, J. A novel murine model to study the impact of maternal depression and antidepressant treatment on biobehavioral functions in the offspring. *Mol Psychiatry* **26**, 6756-6772, doi:10.1038/s41380-021-01145-7 (2021).
